# Real-world clinical and budget impact of semaglutide in type 2 diabetes mellitus in tertiary hospital in Saudi Arabia

**DOI:** 10.3389/fcdhc.2025.1677695

**Published:** 2026-01-27

**Authors:** Hana A. Al-Abdulkarim, Nawaf Salih Alqahtani, Mohammad Al-Shraim, Omar A. Almohammed

**Affiliations:** 1 Ministry of National Guard Health Affairs, King Abdullah International Medical Research Center (KAIMRC), Riyadh, Saudi Arabia; 2 Doctoral School of Applied Informatics and Applied Mathematics, Óbuda University, Budapest, Hungary; 3 Department of Clinical Pharmacy, College of Pharmacy, King Saud University, Riyadh, Saudi Arabia

**Keywords:** diabetes, glycemic control, medical cost, semaglutide, weight loss

## Abstract

**Objective:**

The aim of this research is to evaluate the clinical and economic benefits of semaglutide in patients with type 2 diabetes mellitus (T2DM) in real-world setting in tertiary hospital in Saudi Arabia.

**Methods:**

In this single-center retrospective chart review study, patients were included if they were adults and diagnosed with T2DM for at least one year between January 2017 and December 2023. Patients were excluded if they had previously received glucagon-like peptide-1 receptor agonist (GLP-1RA). The primary outcomes were HbA1c level and Body Mass Index (BMI). Secondary outcomes were the number of outpatient visits, emergency room visits, hospitalization days and total per patient costs.

**Results:**

The study included a total of 186 patients who were initiated on semaglutide. After 12 months on semaglutide, patients’ median HbA1c was reduced from 8.9% [8.4 - 9.9] to 7.8% [7.2 - 8.4] (*p* < 0.0001) and BMI from 34.8 kg/m² [31.2 - 38.5] to 32.6 kg/m² [29.5 - 37.1] (*p* < 0.0001). The number of visits decreased from 3.8 ± 1.6 to 2.9 ± 1.5 visits. Hospitalization days reduced from 7.6 ± 7.2 to 4.6 ± 2.3 days. However, the total cost of care increased from SAR 8,660.6 [6,412.6 - 11,352.3] to 10,434.1 [8,738.4 - 12,553.4]. The total mean additional cost per patient per year was SAR 1,964. Subgroup analyses suggest greater efficacy and higher cost in patients with higher baseline HbA1c and BMI levels.

**Conclusion:**

Semaglutide was associated with significant improvement in glycemic and weight-loss control in adults with T2DM, but this significant change was associated with higher overall cost mainly driven by medication cost.

## Introduction

Type 2 diabetes mellitus (T2DM) is a chronic metabolic disorder characterized by hyperglycemia and insulin resistance, which is primarily caused by beta cell dysfunction in adults (1). Long-term disease can result in severe macrovascular and microvascular complications that have a substantial impact on patient survival and quality of life (2). Indicators of long-term glycemic control in patients with T2DM include glycated hemoglobin (HbA1c), which is regarded as the gold standard way to monitor patients with T2DM (3). According to data from the World Health Organization (WHO), the prevalence of diabetes is very high in Saudi Arabia, where it is ranked second in the Middle East and seventh worldwide (4). In Saudi Arabia, the prevalence of diabetes was estimated to be 17.7% (4.27 million individuals) in 2021 (5, 6). Rapid epidemiological change, urbanization, poor diets, and decreased physical activity in recent decades have all been linked to the alarming rise in number of patients with T2DM in Saudi Arabia (4).

Obesity is a global health issue that affects millions of people worldwide and has serious consequences on both physical and mental well-being (7). It is associated with a higher risk of developing chronic conditions such as heart disease, diabetes, and other comorbidities (7). The WHO defines overweight as having a body mass index (BMI) > 25 kg/m^2^ and obesity by having BMI > 30 kg/m^2^ and describes these conditions as abnormal or excessive fat accumulation that is associated with increased health risk (8). Saudi Arabia notably has an obesity prevalence rate of over 35%, making it one of the highest in the world (8).

Several pharmacological agents are available for the treatment of type 2 diabetes mellitus (T2DM), including glucagon−like peptide−1 receptor agonists (GLP−1RAs) with or without activity on glucose−dependent insulinotropic polypeptide (GIP) (9, 10). Both incretin hormones stimulate insulin secretion after an oral glucose load via the incretin effect to treat T2DM, delay gastric emptying, and inhibit the production of glucagon from pancreatic alpha cells if blood sugar levels are high. Furthermore, GLP-1RA can decrease pancreatic beta-cells apoptosis while promoting their proliferation (9–11). While the most frequently exhibited side effects from GLP-1RA were gastrointestinal (GI) side effects including nausea, vomiting, diarrhea and constipation, as well as injection-site reactions (10).

Semaglutide is a GLP-1RA that was approved for the treatment of T2DM and to reduce the risk of cardiovascular complications in patients with T2DM and cardiovascular disease (12). Clinical trials showed that among adults with overweight or obesity without diabetes, once-weekly subcutaneous semaglutide and lifestyle intervention was associated with substantial, sustained, and clinically relevant mean weight loss of 14.9%, with 86% of participants attaining at least 5% weight loss (13). Moreover, systematic review and meta-analysis evaluated the efficacy and safety of semaglutide for weight loss in 3,613 obese patients without diabetes and concluded that semaglutide was associated with an 11.9% reduction in weight from baseline compared to placebo. However, the risk of GI side effects was 1.6 times higher in the semaglutide group and the risk of serious adverse events such as acute pancreatitis and cholelithiasis was 1.6 times higher in the semaglutide group compared to the placebo group (14).

Diabetes has a significant negative impact on the economy. The total direct cost of diabetes-related treatment in Saudi Arabia was estimated to be 17 billion Riyals annually in 2017. Additionally, it was found that the estimated annual public medical healthcare cost for those with diabetes is ten times that of those without diabetes (6). To preserve glycemic control, lower the risk of diabetes-related complications, and reduce long-term healthcare expenses, the treatment of chronic, progressive disorders like T2D must be promptly intensified. Furthermore, to ensure providing high-quality healthcare with limited resources, further research is required to examine the real-world efficacy and cost implications of new treatment modalities such as semaglutide in a real-world setting.

Several recent studies proved that semaglutide use in Saudi population is associated with favorable benefit in terms of glycemic and weight control (15–17). However, the economic impact has been investigated to a limited extent. The current economic evidence for semaglutide in Saudi Arabia is inconsistent, which poses a challenge for local decision-makers. A previous study explored the cost impact of semaglutide compared to other GLP-1RA. The clinical outcomes data used in the model were derived from clinical trials rather than local real-world settings, which may significantly limit the generalizability of the findings (18). A recent cost-consequence analysis contradicted the previous studies concluding that semaglutide is not superior to other antidiabetic agents in terms of glycemic control while imposing significant additional budget impact (19). In addition, a 5-year budget impact model suggested that introducing semaglutide was budget-neutral to slightly budget-inflating (20). Crucially, these prior economic models either used synthetic patient data from clinical trials or relied on general assumptions, which may not accurately reflect current local prescribing patterns, patient adherence, or complication rates in the Saudi healthcare system. This study is novel because it integrates both the clinical efficacy (HbA1c and BMI reduction) and the financial impact using primary, real-world data from Saudi T2DM patients in a comprehensive budget impact analysis. These inconsistent findings signal the importance of conducting further research to investigate these inconsistencies. In this study, we aim to assess the clinical efficacy of semaglutide in reducing HbA1c and BMI, and its resulting real budget impact in patients with T2DM.

## Methodology

A retrospective, observational chart review was conducted at King Abdulaziz Medical City, Ministry of National Guard Health Affairs (MNGHA), a tertiary care facility in Riyadh, Saudi Arabia with more than 1,500 beds. An institutional review board (IRB) approval was granted by King Abdullah International Medical Research Center (KAIMRC). The study included adult patients aged 14 years and older, male and female, who were previously diagnosed with T2DM. Consistent with the internal policy of King Abdulaziz Medical City, patients aged 14 years and above are classified as adults for clinical care and institutional review board purposes. Patients were eligible for inclusion if they had been on diabetes medications—excluding GLP-1RA—for at least one year prior to initiating semaglutide therapy. The semaglutide utilized in this study was the once-weekly subcutaneous (s.c.) injection, prescribed according to standard clinical practice guidelines for T2DM management at MNGHA. Doses included the approved therapeutic range of 0.5 mg and 1.0 mg weekly. The study period spanned from January 1, 2017 to December 31, 2023. All relevant clinical and economic variables were extracted retrospectively from the electronic medical records. Patients were excluded if they were not eligible to receive care at the MNGHA, had fewer than two follow-up visits per year, had a mean hemoglobin level below 8 g/dL, were diagnosed with hematological disorders, were receiving iron infusion therapy, or had received blood transfusions during the period of semaglutide use. Extracted data included age, gender, HbA1c, BMI, presence of any of the following diseases: hypertension, heart disease, kidney disease, liver disease, dyslipidemia, neurological disease, or pulmonary disease.

The analysis was conducted from the perspective of a public healthcare payer, focusing solely on direct medical costs. A micro-costing approach was used to estimate the total direct cost. Extracted resource use data included medications for example semaglutide, insulins, and other oral antidiabetic, outpatient visits, hospitalization, laboratory tests and semaglutide side effects associated resource use. Then, price per unit was assigned to each resource utilized. Hospitalization and laboratory prices were obtained from the business center’s billing records, while medication prices were sourced from the Oracle system based on acquisition costs in 2023 purchasing transactions. The primary outcomes were HbA1c and BMI. The secondary outcome was the budget impact of introducing semaglutide into the MNGHA formulary. For inclusion in the final analysis, all patients were required to have completed a minimum of 12 months of semaglutide therapy. The primary and secondary endpoints were collected at baseline and at the 12-month after semaglutide initiation.

### Statistical analysis

Baseline patient characteristics were summarized as the mean with standard deviation (SD) for continuous variables with normally distributed data, and frequency with percentage (%) for categorical variables. The normality of data was checked using Shapiro-Wilk test and non-normally distributed data were presented as median (with interquartile range [IQR]). To evaluate statistical significance between the clinical and economical outcomes before and after semaglutide initiation, the Wilcoxon signed-rank test was conducted, as all paired data for the outcome variables were not normally distributed. Data were cleaned and managed using Microsoft Excel (2024) (21). All data were analyzed using SAS software (version 9.4; SAS Institute Inc., Cary, NC, USA).

## Results

### Baseline characteristics

A total of 186 patients met the inclusion criteria for this study. The chart review consisted of individuals with T2DM who had been prescribed semaglutide as part of their treatment plan. At baseline, the mean age of patients was 61.4 ± 10.5 years, and the median for HbA1c was 8.9% [8.4 - 9.9], and for BMI was 34.8 kg/m^2^ [31.2 - 38.5]. Male patients (n=97) accounted for 52.2% of patients. Majority of the included patients had hypertension (83.3%), dyslipidemia (84.4%), or heart disease (59.7%). **Table 1** summarizes the baseline characteristics of the included patients.

**Table 1:** Baseline characteristics.

Variables	Value
Age	61.4 ± 10.5
HbA1c	8.9 [8.4 - 9.9]
BMI	34.8 [31.2 - 38.5]
Gender	
Female	89 (47.8)
Male	97 (52.2)
HTN	155 (83.3)
Heart disease	111 (59.7)
DLP	157 (84.4)
Renal disease	27 (14.5)
Liver disease	4 (2.2)
Pulmonary disease	23 (12.4)
CNS disease	13 (7.0)
Metformin	138 (74.2)
Gliclazide	55 (29.6)
Sitagliptin	78 (41.9)
Pioglitazone	5 (2.7)
Insulin glargine	120 (64.5)
Insulin aspart	105 (56.5)
Insulin degludec	9 (4.8)
Human insulin	14 (7.5)

Numbers are presented as mean ± SD, median [IQR], or N (%).

### Clinical outcomes

After the initiation of patients on semaglutide, there was a significant reduction in HbA1c levels from 8.9% [8.4 - 9.9] before treatment to 7.8% [7.2 - 8.4] representing a mean reduction of 1.4% ± 1.3. This reduction in HbA1c was statistically significant (*p* < 0.0001), suggesting that semaglutide effectively improved glycemic control in these patients.

Additionally, the BMI declined from a median of 34.8 kg/m² [31.2 - 38.5] to 32.6 kg/m² [29.5 - 37.1] after the treatment period, corresponding to a mean reduction of approximately 2.1 ± 2.3 kg/m². This change in BMI was also statistically significant (*p* < 0.0001), indicating that semaglutide had a positive impact on weight management in patients with T2DM. **Table 2** summarizes the clinical outcomes before and after the use of semaglutide.

**Table 2:** Clinical outcomes before and after semaglutide initiation.

Outcomes	Before semaglutide initiation	After semaglutide initiation	*p*-value*
HbA1c, %	8.9 [8.4 - 9.9]	7.8 [7.2 - 8.4]	<0.0001
BMI, kg/m^2^	34.8 [31.2 - 38.5]	32.6 [29.5 - 37.1]	<0.0001

Numbers are presented as median [IQR].

*p-values are from the Wilcoxon signed-rank test.

In terms of safety and tolerability, semaglutide was generally well tolerated among the studied population. The most reported adverse effects were GI in nature, including epigastric pain, nausea or vomiting. Importantly, no major or serious adverse events were observed during the study period, suggesting a favorable safety profile for semaglutide in routine clinical practice.

### Economical outcomes

The total direct cost per patient significantly increased from a median of SAR 8,660.6 [6,412.6 - 11,352.3] before semaglutide initiation to SAR 10,434.1 [8,738.4 - 12,553.4] after initiation (*p*<0.0001). This reflects an average increase of SAR 1,964 per patient annually. The mean cost for semaglutide alone was SAR 3,883 per patient annually, representing 34% of post-initiation medication cost. Clinic visits decreased by 1 visit on average from 3.8 visits per year to 2.9 visits, reducing its associated clinic visit costs from SAR 1,335 to SAR 1,029 per patient. For those who were hospitalized during the study period, hospitalization duration per patient declined by 3 days from 7.6 ± 8.0 to 4.6 ± 2.6 days, contributing to a reduction in mean hospitalization cost from SAR 5,320 ± 5,634 to SAR 3,220 ± 1,825. The proportion of patients visiting emergency room increased slightly from 10.2% to 12.4% of patients; however, the mean emergency room visit cost decreased from SAR 1,402 ± 1,249 to SAR 1,215 ± 1,180. Similarly, the cost of laboratory tests declined from SAR 6,040 ± 2,498 to SAR 4,655 ± 2,369 (**Table 3**). With the introduction of semaglutide, the utilization of insulin and other medications did not significantly change.Subgroup analyses.

**Table 3:** One-year healthcare resource use and costs before and after semaglutide initiation.

Resource	Before semaglutide initiation	After semaglutide initiation
Medication utilization		
Metformin	139 (74.7)	120 (64.5)
Gliclazide	55 (29.6)	37 (19.9)
Sitagliptin	78 (41.9)	103 (55.4)
Pioglitazone	5 (2.7)	8 (4.3)
Insulin glargine, number (%) and units	120 (64.5); 14,974.1 ± 6,977.3	27 (14.5); 14,221.5 ± 6,608.5
Insulin aspart, number (%) and units	105 (56.5); 13,192.1 ± 11,133.7	110 (59.1); 13,196.4 ± 9,506.2
Insulin degludec, number (%) and units	9 (4.8); 9,692.8 ± 4,815.1	11 (5.9); 11,215.5 ± 8,006.9
Human insulin, number (%) and units	14 (7.5); 24,298.6 ± 11,897.9	10 (5.4); 24,236.0 ± 8,856.1
Semaglutide cost per-patient annually, SAR	NA	3,883.1
Cost of semaglutide side effects, SAR	NA	1,556.0 ± 2,600.6
Healthcare utilization		
Number of clinic visit, visits	3.8 ± 1.6	2.9 ± 1.5
Total cost for clinic visits, SAR	1,335 ± 552	1,029 ± 523
ER visits, number of patients and visits	19 (10.2); 2.2 ± 1.9	23 (12.4); 1.9 ± 1.8
Cost per ER visit, SAR	1,402 ± 1,249	1,215 ± 1,180
Hospitalization duration, number and days	5 (2.7); 7.6 ± 8.0	5 (2.7); 4.6 ± 2.6
Cost per hospitalization, SAR	5,320 ± 5,634	3,220 ± 1,825
Laboratory tests cost, SAR	6,040 ± 2,498	4,655 ± 2,369
Total cost per patient, SAR	8,660.6 [6,412.6 - 11,352.3]	10,434.1 [8,738.4 - 12,553.4]

Numbers in the table are presented as Mean ± SD or frequency (percentage).

Subgroup analyses showed that patients with higher baseline BMI (≥40 kg/m2) and HbA1c (≥8.5%) exhibited a larger absolute increase in total healthcare costs. This increase was driven by a greater baseline comorbidity burden and sustained higher utilization of non-drug resources (e.g., hospitalizations and emergency room visits), not the unit price of semaglutide. These patients also showed potentially higher clinical benefits. Patients younger than 40 years had less significant changes in economic and clinical endpoints. Older patients (≥60 years) exhibited the highest post-initiation costs, aligning with their higher baseline health resource utilization. Patients with comorbid conditions (e.g., pulmonary, CNS, and liver diseases) did not have significant improvement in clinical outcomes nor significant difference in cost.

## Discussion

In this retrospective chart review study, we assessed the clinical and economic outcomes of semaglutide at a tertiary hospital in Saudi Arabia. A total of 186 patients with uncontrolled T2DM were included. The analysis showed that semaglutide was associated with a substantial improvement in both HbA1c 8.9% to 7.8% and BMI from 34.8 kg/m² to 32.6 kg/m² without any significant adverse effects. The Diabetes Control and Complications Trial (DCCT) found that a 1% reduction in HbA1C levels was associated with at least a 30% decrease in microvascular complications (22). Thus, HbA1c reduction > 1.5% after the use of semaglutide is considered clinically meaningful.

Additionally, the number of outpatient visits and duration of inpatient stays were reduced significantly leading to reductions in cost. This reduction is in line with the decline in hospital admissions observed in the *post hoc* analysis of the SELECT trial (23). Our results revealed that emergency room visits increased slightly while the mean emergency room visit cost declined. While semaglutide might be associated with reductions in emergency room visits due to its cardiovascular and metabolic benefits, it’s important to note that some patients may experience gastrointestinal side effects, such as nausea and vomiting, which can lead to emergency room visits. However, these instances are relatively rare and often manageable with proper medical oversight. Overall, the total direct cost per patient increased by 21.3% after the initiation of semaglutide which was mainly driven by the cost of semaglutide. In addition, our sub-analyses showed that older patients, and those with higher baseline HbA1c and BMI levels, had the highest absolute post-initiation total healthcare costs. This finding is consistent with their greater underlying disease severity and associated comorbidity burden, leading to higher consumption of non-drug resources, such as specialized clinic visits and inpatient care, which compounded the fixed cost of semaglutide. In addition to these findings, we observed an unexpected increase in sitagliptin use following the initiation of semaglutide. As GLP-1 receptor agonists and DPP-4 inhibitors share overlapping incretin-based mechanisms and are not recommended for concurrent use, this pattern likely reflects unintentional therapeutic duplication rather than a deliberate clinical decision. This observation suggests an opportunity to enhance system-wide awareness of current guideline recommendations and ensure appropriate therapy optimization. Strengthening routine medication review processes may help reduce unnecessary duplication and support more efficient use of healthcare resources.

Our results are broadly in line with those of previous local efficacy and safety studies that proved the efficacy and safety of semaglutide in real-world setting in Saudi Arabia (15–17). However, our findings differ from the results demonstrated by an earlier recently published cost-consequences study conducted locally (18). Their analysis suggested no difference in HbA1c levels and hospitalization with the use of semaglutide. Moreover, their study estimated a much higher annual per-patient cost than ours (SAR 21,875.36 ± 1,899.56 vs SAR 11,156.2 ± 3,407.2).

The limitations of the study are inherently associated with the characteristics of retrospective pre/post studies. Bias from poor data quality and underreporting of resource use can result in underestimating actual costs. Another limitation is the inability to control for confounding variables due to the absence of a control arm. Moreover, the brief follow-up duration may be insufficient to capture long-term benefits and costs. Future studies with extended follow-up periods could be valuable in evaluating the sustainability of the effects and the long-term advantages in mitigating complications, thereby reducing costs. Moreover, further research is required to assess the long-term cost-effectiveness of semaglutide, encompassing both the long-term costs and benefits of the drug.

## Conclusion

This retrospective study showed that semaglutide is effective in reducing both HbA1c and BMI and is a well-tolerated treatment option for patients with T2DM, offering dual benefits of glycemic control and weight reduction. While the direct pharmaceutical costs increased -primarily driven by the cost of semaglutide, reductions in clinic visits, hospitalization duration, and associated healthcare services contributed to a partial offset of the overall budget impact. The trends suggest semaglutide potential role in optimizing long-term health outcomes and reducing major complication-related healthcare burdens.

## Data Availability

The raw data supporting the conclusions of this article will be made available by the authors, without undue reservation.
